# The Potential of Ectoine as a Brain Anti-Aging Agent in Rat Model of d-Galactose-Accelerated Aging May Be Mediated Through Crosstalk Between Redox/Mitochondrial Homeostasis/Autophagic/Apoptotic Pathways

**DOI:** 10.1007/s12035-025-05451-x

**Published:** 2025-12-08

**Authors:** Aisha H. A. Alsenousy, Marwa B. Bakir, Mohammed S. Zommara, Hanan A. Edres, Wael S. Darwish, Rania M. H. M. Eid, Maher A. Kamel

**Affiliations:** 1 Department of Biochemistry, Medical Research Institute, University of Alexandria, 165 El-Horeya Rd, Al Ibrahimeyah Qebli WA Al Hadrah Bahri, Qesm Bab Sharqi, 21561 Alexandria, Egypt; 2 Medical Education Department, Faculty of Medicine, Najran University, Najran, Saudi Arabia; 3 Department of Internal Medicine, Faculty of Medicine, University of Alexandria, Alexandria, Egypt; 4 Department of Biochemistry, Faculty of Veterinary Medicine, Alexandria University, Alexandria, Egypt; 5 Department of Basic Science, College of Dentistry, Al Maaqal University, Al Basrah, 61003 Iraq; 6 Department of Physiology, Faculty of Medicine, Aswan University, Aswan, 81528 Egypt

**Keywords:** Aging, Apoptosis, Autophagy, D-galactose, Ectoine, MiR-124, Mitochondrial homeostasis, Neuroinflammation, Neuroprotection, Oxidative stress

## Abstract

Aging has emerged as a prominent area of academic inquiry. Brain aging is a complex physiological process characterized by features such as enhanced apoptosis, oxidative stress, neuroinflammation, mitochondrial dysfunction, and impaired autophagy. Currently, effective preventative or therapeutic approaches for age-related neurodegenerative disorders remain elusive. Ectoine, a naturally occurring compatible solute, possesses diverse applications in biological engineering, cosmetics, medicine, and the food industry. Ectoine is reported to exhibit anti-inflammatory, antioxidant, and anti-apoptotic properties, making it a potential anti-aging agent. Consequently, the present study investigated the potential neuroprotective effects of Ectoine against d-galactose (D-gal)-induced brain aging. Accelerated aging was induced by subcutaneous injection of D-gal. Rats were subsequently divided into a control group, an aged group, and Ectoine-supplemented groups, receiving daily doses of 10, 20, and 40 mg/kg, respectively. Our findings revealed that Ectoine effectively and dose-dependently protected against D-gal-induced brain aging by inhibiting oxidative stress, enhancing the antioxidant system, decreasing neuroinflammation, restoring autophagy and mitochondrial homeostasis, and inhibiting apoptosis. Furthermore, Ectoine significantly restored the expression of miR-124 and its target genes; however, this effect is correlative and warrants further mechanistic validation. Additionally, while Ectoine’s neuroprotective effects were observed at the tissue level, its cell-type specificity remains to be determined. These findings suggest that Ectoine may exert multi-pathway neuroprotective effects in brain aging. However, the current data are exploratory and warrant further validation to define causality and translational applicability.

**Graphical Abstract:**

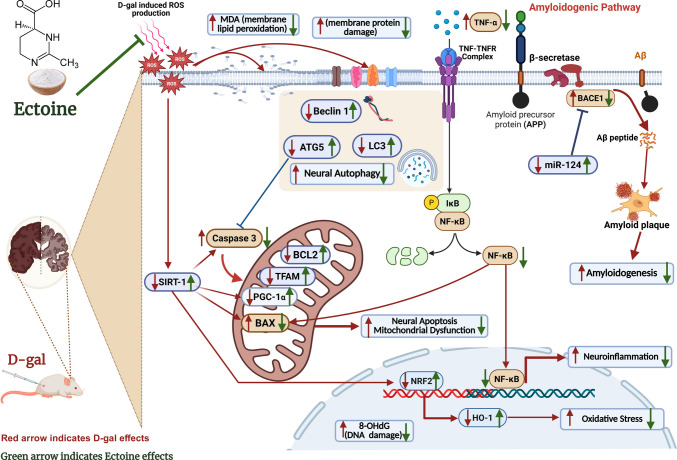

## Introduction

Aging is a complex biological process characterized by a progressive decline in physiological function, leading to increased susceptibility to disease and mortality [1]. The aging brain exhibits structural, molecular, and functional changes, which collectively result in cognitive decline and an elevated risk of neurodegenerative diseases [2]. Molecular hallmarks of brain aging encompass telomere shortening, oxidative stress resulting from reactive oxygen species (ROS) accumulation, neuroinflammation propelled by chronic microglial activation, dysregulated mitochondrial homeostasis, impaired autophagy, and the pathological accumulation of misfolded proteins like amyloid-beta (Aβ) and tau, which are central to Alzheimer’s disease (AD) and other neurodegenerative conditions. Critically, these processes are highly interconnected; oxidative stress and mitochondrial dysfunction, for instance, commonly exacerbate neuroinflammation and protein misfolding, thereby establishing a vicious cycle that accelerates brain aging [3, 4].

A key feature of brain aging is the dysregulation of the amyloidogenic pathway, which involves the abnormal processing of amyloid precursor protein (APP) by β-secretase (BACE1). This leads to the accumulation of beta-amyloid (Aβ) peptides (e.g., Aβ1–42), which subsequently aggregate into toxic oligomers and plaques. These aggregates disrupt synaptic function, induce oxidative stress, and trigger neuroinflammation. This pathway is central to Alzheimer’s disease (AD) pathology and is additionally implicated in age-related cognitive decline [5]. Furthermore, microRNA-124 (miR-124) influences this pathway by directly targeting BACE1 [6].

During aging, the brain undergoes significant changes in its redox status, primarily due to an imbalance between reactive oxygen species (ROS) generation and antioxidant defenses, which results in oxidative stress [7]. Brain tissues are particularly susceptible to ROS-mediated damage because of their high abundance of polyunsaturated fatty acids, relatively diminished antioxidant levels, and increased oxygen demand [8]. This enhanced ROS generation, coupled with decreased antioxidants in the aged brain, exacerbates redox imbalance and contributes to neuroinflammation and cellular damage [9, 10]. This oxidative stress is closely linked to dysregulation of the crucial redox signaling pathway, nuclear factor-erythroid 2-related factor 2 (NRF2), which acts as a transcription factor vital for maintaining cellular redox balance and modulating inflammatory responses. Under stress conditions, NRF2 is stabilized and translocates to the nucleus, where it binds to the antioxidant response element (ARE) to regulate the expression of protective genes [11]. However, in the aged brain, the NRF2 pathway often exhibits reduced responsiveness, thereby impairing adaptive cellular responses to oxidative stress [12].

Mitochondria are essential for brain function, primarily by generating ATP through oxidative phosphorylation to satisfy the high energy demands of neurons, particularly for synaptic activity and the maintenance of ion gradients. Beyond energy production, they also play a crucial role in regulating calcium homeostasis and reactive oxygen species (ROS) signaling [13]. Mitochondrial homeostasis refers to the dynamic equilibrium of mitochondrial quality, quantity, and function within cells. This involves processes designed to ensure mitochondria remain healthy, fully functional, and appropriately distributed, thereby enabling cells to meet their energy demands and adapt effectively to stress [14]. A key mechanism contributing to mitochondrial homeostasis is mitochondrial biogenesis, which is primarily governed by the mitochondrial transcription factor A (TFAM)/peroxisome proliferator-activated receptor co-activator 1α (PGC-1α) pathway [15, 16], Mitochondrial homeostasis relies on several key mechanisms: mitochondrial biogenesis (which was likely discussed previously, as this paragraph starts mid-list); mitochondrial fusion (merging) and fission (division), which together maintain mitochondrial morphology, distribution, and function, with key regulators including cytosolic dynamin-related protein 1 (Drp1) for fission and Mitofusin 1 and 2 (Mfn1/2) for fusion [[17]; and mitophagy, a selective degradation process that prevents the accumulation of dysfunctional organelles [14]. Mitochondrial dysfunction is a recognized hallmark of aging, and the accumulation of mitochondrial DNA damage, overproduction of reactive oxygen species (ROS), and impaired quality control mechanisms collectively contribute to brain aging [18].

Autophagy and apoptosis are essential cellular housekeeping and tissue survival mechanisms, and mitochondria are notably central regulators of the crosstalk between these two processes [19]. Mitochondria control the intrinsic apoptotic pathway through the action of Bcl-2 family proteins, which include both anti-apoptotic (e.g., Bcl-2 and Bcl-xL) and pro-apoptotic (e.g., Bax) members. The complex process of autophagy involves a coordinated network of various proteins, including microtubule-associated protein 1 light chain 3 (LC3) and Beclin-1, both of which are critical for its initiation. Autophagy-related gene 5 (ATG5) is an essential protein involved in the formation of autophagosomes and the subsequent progression of autophagy [20]. Impaired autophagy is considered one of the hallmarks of aging [21]. Studies have reported that Bcl-2 can repress Beclin 1-dependent autophagy, a process whose inhibition may accelerate aging [22]. Furthermore, Sirtuin 1 (SIRT1), a conserved sirtuin, regulates various cellular processes and exerts neuroprotective effects in diverse central nervous system disorders, primarily by activating the PGC-1α/NRF2 signaling pathways [23].

Despite advancements in our understanding of brain aging, effective pharmacological therapies remain limited and are frequently associated with significant adverse effects. This underscores the critical need for novel, safe, and naturally derived therapeutic interventions [24]. One of the promising compounds is ectoine, a naturally occurring osmolyte produced by extremophilic microorganisms. Ectoine has attracted interest due to its ability to defend against environmental stressors as drought and UV radiation. [25]. While Ectoine’s antioxidant and anti-inflammatory effects in dermatological applications, as well as in vitro potential to inhibit Aβ aggregation, are extensively documented, research has predominantly focused on topical or inhalation routes of administration [26, 27]. Consequently, its efficacy as an orally administered therapeutic agent, particularly concerning brain aging, remains largely unexplored. Specifically, no prior studies have investigated the preventive effects of oral Ectoine within the d-galactose-induced brain aging model, a critical research gap that the present study directly addresses.

This study investigates the neuroprotective potential of orally administered Ectoine as an anti-aging agent in a d-galactose-induced rat model of accelerated brain aging. We specifically focus on its modulation of critical cellular and molecular pathways implicated in aging, including redox homeostasis (e.g., NRF2 pathway), mitochondrial homeostasis (e.g., PGC-1α/TFAM, DRP1/MFN2, and mitophagy), autophagy (e.g., Beclin-1, LC3, ATG5), apoptosis (Bcl-2/Bax), and the amyloidogenic pathway (e.g., BACE1/Aβ). Exploring the intricate crosstalk among these pathways, the present investigation aims to provide novel insights into Ectoine’s systemic neuroprotective mechanisms. This approach distinguishes our findings from prior research that focused on Ectoine’s topical applications or in vitro effects. Moreover, the employment of oral administration significantly enhances the translational potential of Ectoine as a non-invasive therapeutic strategy for age-related cognitive decline, thereby addressing a critical unmet need in brain aging research.

## Materials and Methods

### Experimental Animals

A total of 25 male Wistar rats, aged 2–3 months, were obtained from the Animal Facility, Medical Technology Center, Medical Research Institute, Alexandria University, Egypt. Animals were housed in groups of five per cage under controlled environmental conditions, including a constant temperature of 23 °C and a 12-h light/12-h dark cycle. All animals received ad libitum access to standard rodent chow and water and were maintained under standard hygienic conditions.

### Ethical Statement

The study protocol was approved by the Institutional Animal Care and Use Committee (IACUC) of Alexandria University (protocol number AU01224111031). All experimental procedures were conducted in strict accordance with the guidelines for the care and use of laboratory animals (USA National Institutes of Health Publication No. 80–23, updated 1996). Every effort was made to minimize animal discomfort and suffering throughout the experimental period.

### Induction of D-gal Accelerated Aging

D-gal accelerated aging was induced in young albino rats (approximately 2 months old and weighing 120–140 g) by daily subcutaneous injection of D-gal at a dosage of 200 mg/kg/day for 42 days [28].

### Experimental design

Animals were randomly assigned to three main experimental groups for 42 days: Group I (control, *n* = 5)—received daily subcutaneous (SC) injections of physiological saline; Group II (d-galactose-induced aging, *n* = 5)—received daily SC injections of d-galactose (200 mg/kg/day); Group III (d-galactose + Ectoine treatment, *n* = 15 total)—these rats received daily SC injections of d-galactose (200 mg/kg/day) and were concurrently administered oral Ectoine. This group was further subdivided into three Ectoine dose subgroups (*n* = 5 per subgroup): 10 mg/kg/day, 20 mg/kg/day, and 40 mg/kg/day. Ectoine doses were selected based on previous research [29], demonstrating its efficacy and safety in non-neurological models. These doses were adjusted for oral administration in rats, given Ectoine’s high solubility and lack of toxicity.

After day 42 of the treatment period and after overnight fasting, all rats were sacrificed by deep anesthesia using isoflurane inhalation. The brain tissues were dissected out and the cortical tissues were quickly removed, perfused and washed with saline, and were divided into four aliquots; the first one was used for total RNA extraction to assess gene expression, the second aliquot was homogenized in RIPA buffer in a ratio of 1:9 and centrifuged at 10,000 × g for 10 min at 4 °C and the supernatant was stored in aliquots was used for the assessment of total protein, malondialdehyde (MDA), glutathione (GSH), neurotransmitters, inflammatory markers, and antioxidants, the third aliquot for DNA extraction to assess 8-hydroxy deoxy-guanosine (8-OHdG) and mitochondrial DNA copy number (mtDNA-CN), and the last aliquot was fixed in neutral buffered formaldehyde for histological studies.

### Protein Assessments By ELISA

From the prepared tissue supernatant, levels of NRF2, heme-oxygenase-1 (HO-1), SIRT1, TNF-α, NF-κB, BAX, BCL-2, Aβ1–42, dopamine, and acetylcholine (ACh) were assessed. These measurements were performed using commercially available rat-specific enzyme-linked immunosorbent assay (ELISA) kits (Chongqing Biospes Co., Ltd., Cat. No. BYEK3218) according to the manufacturer’s instructions. Before quantification, the total protein concentration in each supernatant sample was determined using the Lowry method [30].

### Caspase-3 Activity By Colorimetric Assay

Caspase-3 enzymatic activity was determined using the Caspase-3 Assay Kit (Elabscience, USA). This kit utilizes a chromogenic substrate, acetyl-Asp-Glu-Val-Asp p-nitroanilide (Ac-DEVD-pNA), which consists of a caspase-3 sequence-specific peptide conjugated to the yellow chromophore p-nitroaniline (pNA). Upon cleavage by active caspase-3, the pNA molecule is liberated. The resulting increase in absorbance at 405 nm, directly proportional to caspase-3 activity, was then measured spectrophotometrically according to the manufacturer’s instructions.

### DNA Content of 8-OH-2-Deoxyguanosine (8-OHdG) as the Index of Oxidative DNA Damage

The determination of 8-OHdG levels in DNA commenced with the extraction of genomic DNA using a DNeasy kit (Qiagen, Germany). The isolated DNA was subsequently subjected to enzymatic digestion with nuclease P1. The resulting DNA hydrolysate was then used to determine 8-OHdG concentrations using a commercially available 8-OH-dG ELISA kit (ab201734, Abcam, Cambridge, UK), strictly adhering to the manufacturer’s instructions.

### Determination of Malondialdehyde (MDA) Using Thiobarbituric Acid Reactive Substances (TBARS) Reaction

Malondialdehyde (MDA) was determined according to the method of Draper and Hadley. The brain cortical tissue samples were heated with thiobarbituric acid at acidic pH. The resulting pink chromogen has a maximal absorbance of 532 nm [31].

### Determination of Glutathione (GSH) and Glutathione Disulfide (GSSG)

Griffith et al. described an enzymatic technique for measuring total glutathione and GSSG concentration, which was then utilized to determine reduced GSH. This technique involves oxidizing GSH with 5,5′-dithiobis-(2-nitrobenzoic acid) (DTNB) to produce GSSG and 5-thio-2-nitrobenzoic acid (TNB). Glutathione reductase and nicotinamide adenine dinucleotide phosphate (NADPH) enzymatically reduce oxidized GSSG to yield GSH, which then reacts again. The continuous production of TNB is measured spectrophotometrically at 412 nm, with its rate directly proportional to the total glutathione concentration (GSH + GSSG) in the sample [32].

### Assay of Mitochondrial DNA-Copy Number (mtDNA-CN)

The mtDNA copy number relative to nuclear DNA was assayed using real-time polymerase chain reaction (qPCR) [33]. The genomic DNA was amplified using specific primer pairs for the mtDNA sequence (NC_040919.1, Forward; AATGGTTCGTTTGTTCAACGATT and Reverse; AGAAACCGACCTGGATTGCTC) and the nuclear PGC-1α gene (NC_086032.1, Forward; ATGAATGCAGCGGTCTTAGC and Reverse; AACAATGGCAGGGTTTGTTC) using qPCR under the following conditions. 98 °C for 2 min, then 45 cycles of 98 °C for 10 s and 55 °C for 30 s. The relative amount of mtDNA signal to nuclear DNA signal was determined. The nuclear gene was utilized to measure nuclear DNA (nDNA); therefore, the mtDNA-CN was normalized to the cell’s nDNA using the equation:

$$R=2^{-\Delta Ct},\;where\;\Delta Ct=Ct_{mtDNA}-Ct_{nuclear}.$$

### Cortical Gene Expression Using qPCR

The total RNA was extracted using the RNeasy Mini Kit (Qiagen, Germany) following the manufacturer’s instructions. The purity and concentration of the extracted RNA were determined using a Nanodrop. RNA was reverse-transcribed using TOPscript™ RT DryMIX kit (dT18/dN6 plus) (En-zynomics, Korea), according to the manufacturer’s instructions. Beclin 1, LC3B, ATG5, BACE1, PGC-1α, TFAM, MFN2, and DRP1 were quantified in cDNA using CFX Maestro™ Software (Bio-Rad, USA) and QuantiNova™ SYBR® Green PCR Kit (Qiagen, Germany). The conditions for thermal cycling were initially denaturation at 95 °C for 10 min, followed by 45 cycles of 95 °C for 20 s (denaturation), 55 °C for 20 s (annealing), and 70 °C for 15 s (extension). The 18S rRNA housekeeping gene was used as an internal reference for normalization. The primers used to determine rat genes are shown in Table 1. The 2^−ΔΔCt^ method was used to evaluate the relative change in mRNA expression within samples.

**Table 1:** Primer sequences

Gene	Accession number	Primer sequence	
Beclin 1	NM_001034117.1	F:	TTGGCCAATAAGATGGGTCTGAA
		R:	TGTCAGGGACTCCAGATACGAGTG
LC3B	NM_022867.2	F:	CAGGATCCATGCCGTCCCAGAAGACC
		R:	GTCCCTTTTTGCCTTGGTAG
ATG5	NM_001014250.1	F:	CAGAAGCTGTTCCGTCCTGT
		R:	CCGTGAATCATCACCTGGCT
BACE1	NM_019204.2	F:	GCATGATCATTGGTGGTATC
		R:	CCATCTTGAGATCTTGACCA
PGC-1α	NM_031347.1	F:	GTGCAGCCAAGACTCTGTATGG
		R:	GTCCAGGTCATTCACATCAAGTTC
TFAM	NM_031326.2	F:	CCCTGGAAGCTTTCAGATACG
		R:	AATTGCAGCCATGTGGAGG
MFN2	NM_130894.4	F:	GCCAGCTTCCTTGAAGACAC
		R:	GCAGAACTTTGTCCCAGAGC
DRP1	NM_053655.3	F:	AATCCTAATTCCATTATCCTCGCT
		R:	ACCAGTAGCATTTCTAATGGC
18S rRNA	NR_046237.2	F:	GTAACCCGTTGAACCCCATT
		R:	CAAGCTTATGACCCGCACTT

### Cortical miR-124 Expression Using qPCR

MiR-124 expression in the cortex tissue was assayed using TaqMan® miR-124 (Thermo Fisher Scientific, Cat. no. 4427975, ID: 002893) kit and using U6 as a reference gene (cat no. 4427975, ID: 001973). Quantitative PCR was performed with an initial denaturation at 95 °C for 10 min, followed by 45 cycles of 95 °C for 5 s (denaturation), 55 °C for 15 s (annealing), and 60 °C for 15 s (extension). Amplification, data acquisition, and analysis were performed on a CFX96 Touch Deep Well Real-Time PCR Detection System (Bio-Rad Laboratories, California, USA). The values of the threshold cycle (Ct) were determined by CFX Maestro™ Software version 1.1 (Bio-Rad Laboratories, California, USA). The relative change in miR-124 in samples was determined using the 2^−ΔΔCt^ method and normalized to the reference U6.

### Histopathological Assessment

Brain samples were harvested and immediately fixed in 10% neutral buffered formalin (NBF) for 48 h. Following fixation, samples underwent standard paraffin embedding. Four-micrometer-thick sections were then cut and stained with Hematoxylin and Eosin (H&E) as previously described [34].

### Statistical Analysis

Statistical analyses were performed using GraphPad Prism software (version 5.0). Data are presented as mean ± standard deviation (SD). The normally distributed parameters were assessed using the Kolmogorov–Smirnov test. One-way analysis of variance (ANOVA) was conducted, followed by Bonferroni’s post hoc test, to compare mean values among the experimental groups (control, untreated, and treated).

Given the modest sample size in our study (*n* = 5), Cohen’s *d* effect sizes and their corresponding 95% confidence intervals (CIs) were analyzed for key comparisons. This approach provided a more nuanced understanding of the magnitude and precision of the observed effects, complementing traditional *p*-values. Our analysis focused on comparisons between the control group, the aged group, and the aged group treated with the highest dose of Ectoine (40 mg/kg), as these represented the most critical comparisons for the study’s objectives. Effect sizes were deemed meaningful if they were large (Cohen’s *d* > 0.8) and their 95% CIs did not cross the zero line.

## Results

### Effect of Ectoine on Cortical Neurotransmitters of Aged Rats

**Fig. 1:**
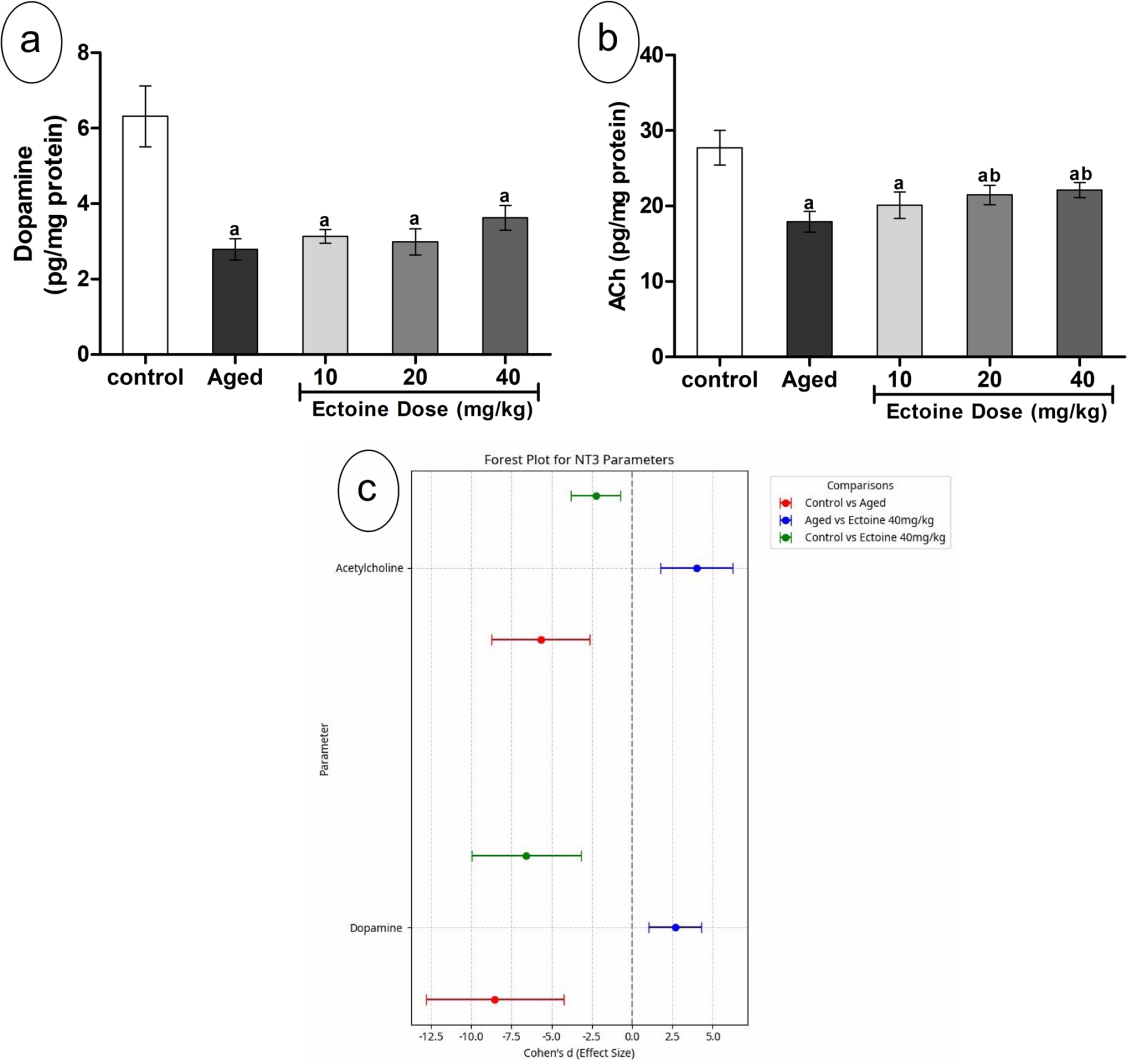
The levels of (**a**) dopamine and (**b**) acetylcholine in the brain cortex of control rats, aged rats, and aged rats treated with different doses of Ectoine (data are presented as mean ± SD. *n* = 5. a: significantly different vs. control rats, b: significantly different vs. aged rats, c: significantly different vs. 10 mg/kg Ectoine-treated aged rats, d: significantly different vs. 20 mg/kg Ectoine-treated aged rats by ANOVA followed by Bonferroni post hoc test at *p* < 0.05) (**c**) Effect sizes (Cohen’s *d* ± 95% CI) of aging and Ectoine supplementation on the dopamine and acetylcholine

Cortical dopamine and ACh content significantly declined in aged rats compared to controls. Ectoine co-supplementation, across increasing doses, had no significant effect on cortical dopamine levels (Fig. 1(a)). In contrast, the highest Ectoine doses (20 and 40 mg/kg) significantly elevated cortical ACh content compared to the aged group, although these levels remained considerably lower than those in controls (Fig. 1(b)).

For both dopamine and ACh, the comparison between control and aged groups revealed large effect sizes, indicating a substantial and clinically meaningful difference attributed to the aging model, thereby reinforcing its severity (Fig. 1(c)). Furthermore, large effect sizes were observed when comparing aged rats to those treated with 40 mg/kg Ectoine. Similarly, the comparison between control and Ectoine (40 mg/kg)-treated groups also showed large effect sizes, demonstrating that while Ectoine significantly ameliorated these parameters, complete restoration to control levels was not achieved (Fig. 1(c)).

### Effect of Ectoine on Cortical Oxidative Stress Markers

**Fig. 2:**
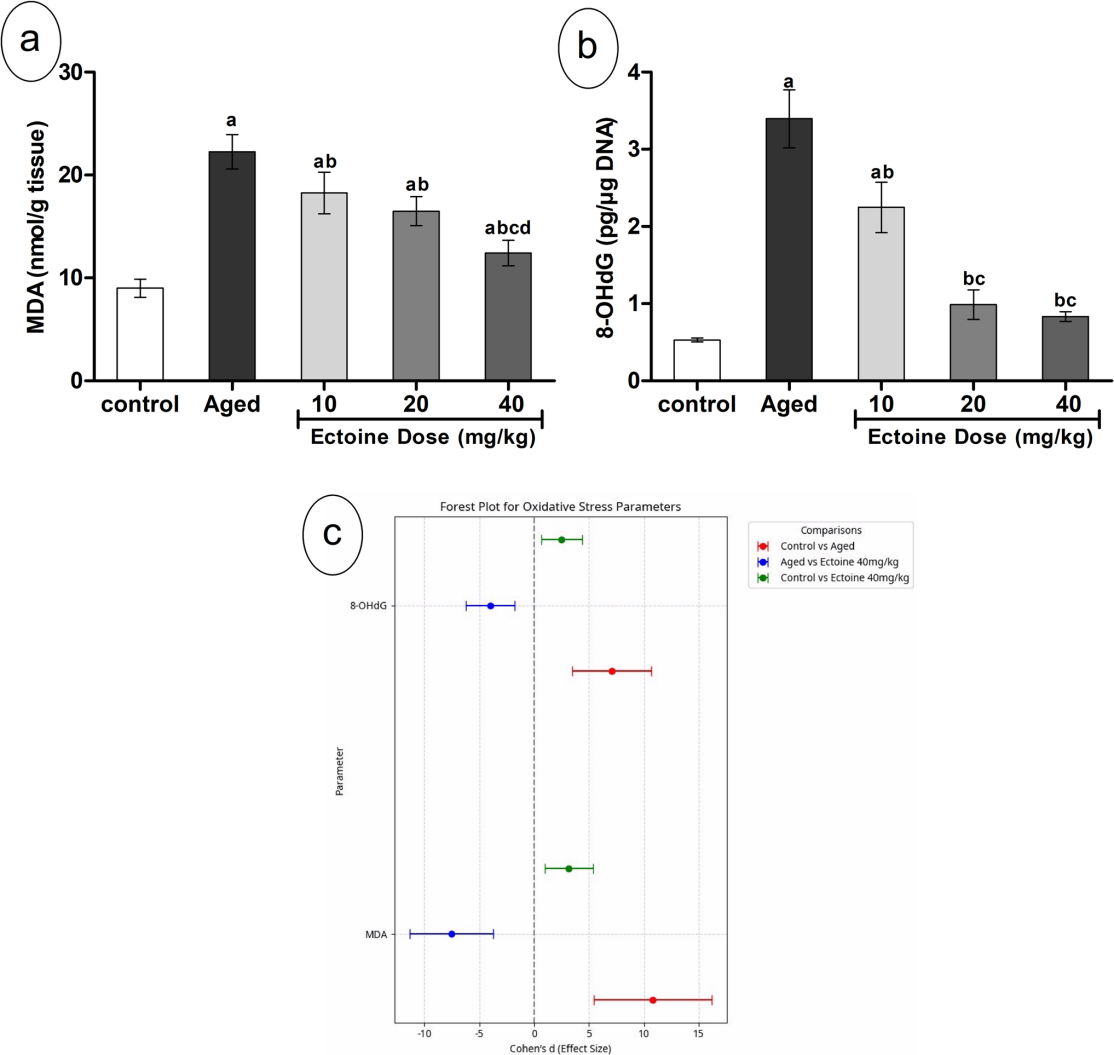
The levels of oxidative stress parameters; (**a**) MDA and (**b**) 8-OHdG in the brain cortex of control rats, aged rats, and aged rats treated with different doses of Ectoine (Data are presented as mean ± SD. *n* = 5. a: significantly different vs. control rats, b: significantly different vs. aged rats, c: significantly different vs. 10 mg/kg Ectoine-treated aged rats, d: significantly different vs. 20 mg/kg Ectoine-treated aged rats by ANOVA followed by Bonferroni post hoc test at *p* < 0.05). (**c**) Effect sizes (Cohen’s *d* ± 95% CI) of aging and Ectoine supplementation on the MDA and 8-OHdG. (Abbreviations: MDA, malondialdehyde; 8-OHdG, 8-hydroxy-2′-deoxyguanosine)

Cortical levels of MDA, a key marker of lipid peroxidation, and 8-hydroxy-2′-deoxyguanosine (8-OHdG), an indicator of oxidative DNA damage, were significantly elevated in aged rats compared to controls (Fig. 2(a, b)). Ectoine supplementation significantly and dose-dependently reduced both MDA and 8-OHdG levels compared to aged rats (Fig. 2(a, b)). Despite this improvement, the levels of both markers remained significantly higher than those observed in the control group.

Consistent with these findings, the comparison between aged and control groups revealed large effect sizes for both MDA and 8-OHdG. Furthermore, Ectoine treatment at 40 mg/kg resulted in large effect sizes when comparing the aged group to the treated group, indicating a substantial amelioration of these oxidative damage markers. This amelioration, although significant, did not result in complete normalization to control levels, as further evidenced by the effect sizes observed in the comparison between the control group and the Ectoine 40 mg/kg group (Fig. 2(c)).

### Effect of Ectoine on NRF2/HO-1 and SIRT1 in the Brain Cortex of Aged Rats

**Fig. 3:**
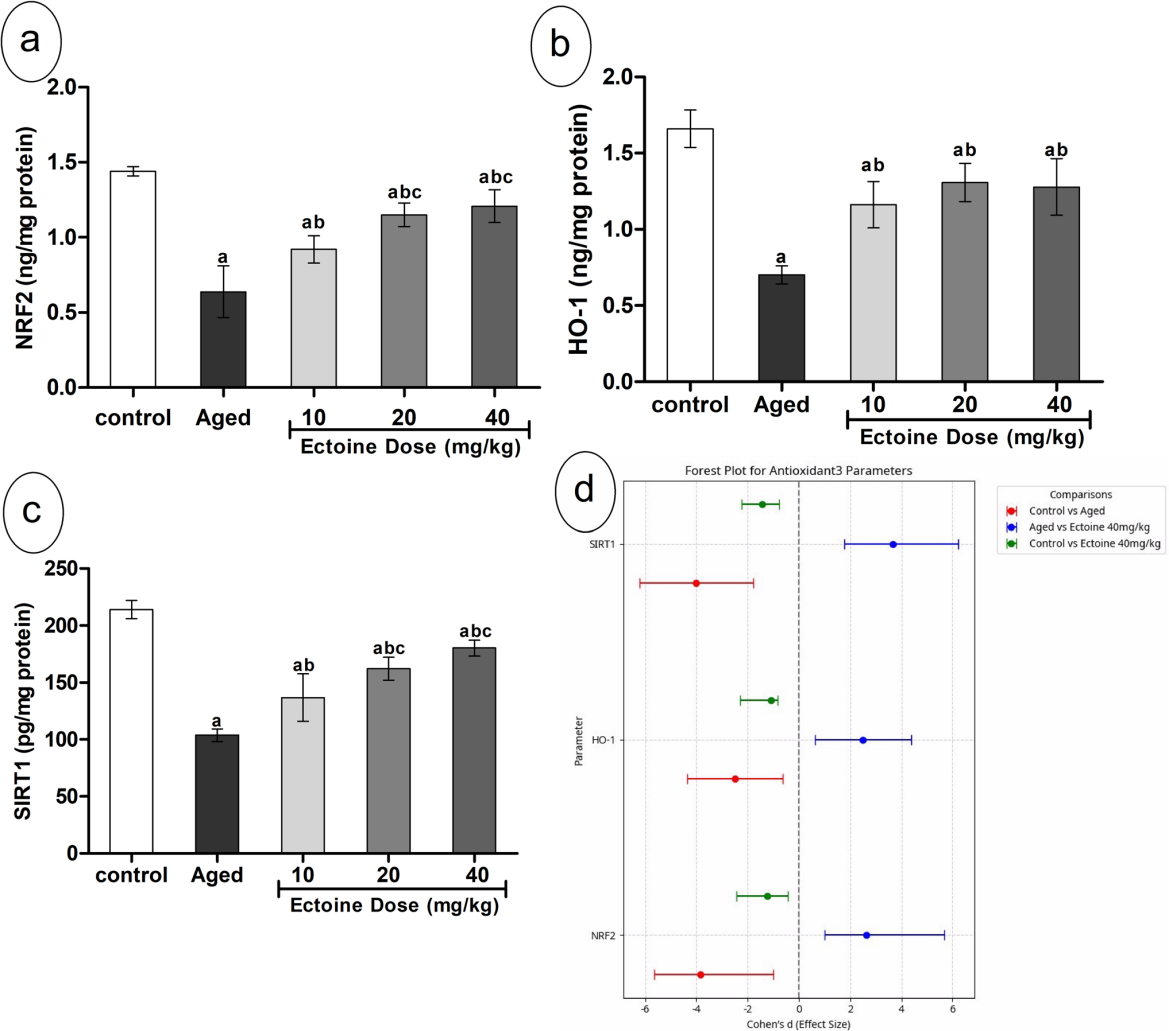
Antioxidant parameters; (**a**) NRF2, (**b**) HO-1, and (**c**) SIRT-1 in the brain cortex of control rats, aged rats, and aged rats treated with different doses of Ectoine (Data are presented as mean ± SD. *n* = 5. a: significantly different vs. control rats, b: significantly different vs. aged rats, c: significantly different vs. 10 mg/kg Ectoine-treated aged rats, d: significantly different vs. 20 mg/kg Ectoine-treated aged rats by ANOVA followed by Bonferroni post hoc test at *p* < 0.05). (**d**) Effect sizes (Cohen’s *d* ± 95% CI) of aging and Ectoine supplementation on the NRF2, HO-1, and SIRT-1. (Abbreviations: NRF2, nuclear factor erythroid 2–related factor 2; HO-1, Heme Oxygenase 1; SIRT1, Sirtuin1)

Cortical content of NRF2 and HO-1, key components of the NRF2/HO-1 system, exhibited a significant decline of approximately 55% in aged rats compared to controls. Co-supplementation with Ectoine significantly and dose-dependently increased the cortical content of both NRF2 and HO-1 compared to aged rats. No significant differences were observed between the 20 mg/kg and 40 mg/kg Ectoine doses. Despite treatment with the highest Ectoine dose, cortical NRF2 and HO-1 content remained significantly lower than that observed in controls (Fig. 3(a, b)). Cortical SIRT1 content exhibited a similar pattern of changes as NRF2/HO-1. Its level significantly declined in the aged rats and was dose-dependently elevated in the Ectoine-protected groups (Fig. 3(c)). When comparing aged rats to controls, NRF2, HO-1, and SIRT1 all demonstrated large effect sizes, indicating a significant negative impact of aging on these parameters. Conversely, the Ectoine 40 mg/kg group showed significant recovery, evidenced by large effect sizes (Fig. 3(d)).

### Effects of Ectoine on the GSH System in the Brain Cortex

In d-galactose-induced aged rats, cortical content of total glutathione (tGSH), reduced glutathione (GSH), and the redox ratio (GSH/GSSG) significantly declined, while oxidized glutathione (GSSG) content was significantly elevated compared to control animals. Ectoine co-supplementation in d-galactose-treated rats significantly and dose-dependently increased tGSH, GSH, and the redox ratio, simultaneously decreasing GSSG content, compared to the untreated aged group. Notably, tGSH, GSH, and GSSG content achieved complete normalization with the highest Ectoine dose (40 mg/kg), as detailed in Table 2.

**Table 2:** Glutathione system parameters in different studied groups

Parameter Groups		tGSH	GSH	GSSG	GSH/GSSG
Control		33.43 ± 2.22	29.77 ± 2.19	1.83 ± 0.09	16.32 ± 1.39
Aged rats		23.39 ± 1.51^a^	16.96 ± 1.08^a^	3.22 ± 0.29^a^	5.29 ± 0.39^a^
Ectoine-treated group	10 mg/kg	24.90 ± 1.59^a^	19.65 ± 0.93^a^	2.62 ± 0.50^ab^	7.66 ± 1.15^ab^
	20 mg/kg	26.06 ± 1.79^a^	21.59 ± 1.41^b^	2.24 ± 0.25^b^	9.71 ± 0.89^abc^
	40 mg/kg	29.75 ± 2.17^bc^	25.77 ± 2.02^bcd^	1.99 ± 0.14^bc^	12.97 ± 1.02^abcd^

Data are presented as mean ± SD. *n* = 5. *tGSH* total glutathione, *GSH* reduced glutathione, *GSSG* oxidized glutathione. ^a^Significantly different vs. control rats. ^b^Significantly different vs. aged rats. ^c^Significantly different vs. 10 mg/kg Ectoine-treated aged rats. ^d^Significantly different vs. 20 mg/kg Ectoine-treated aged rats by ANOVA followed by Bonferroni post hoc test at *p* < 0.05, and 95% CI

**Fig. 4:**
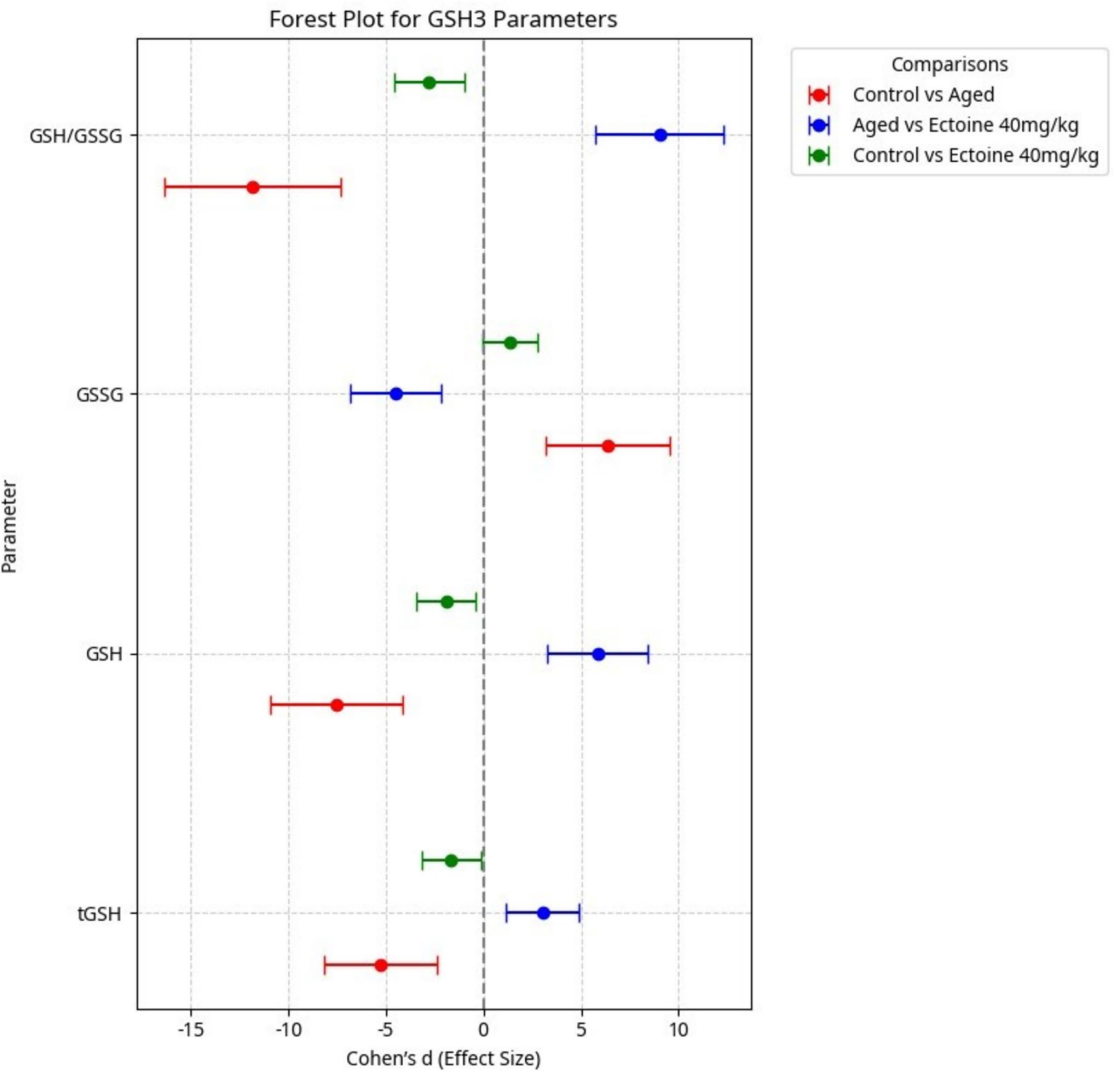
Effect sizes (Cohen’s *d* ± 95% CI) of aging and Ectoine supplementation on the components of glutathione system in brain cortex

Analysis of the Forest plot (Fig. 4) for all glutathione parameters revealed large effect sizes in the comparison between control and aged groups. This indicates a substantial and clinically meaningful difference in glutathione system parameters due to d-galactose-induced aging. Furthermore, the comparison between aged rats and those treated with 40 mg/kg Ectoine also yielded large effect sizes, underscoring the potent ameliorative impact of Ectoine on the d-galactose-induced impairments in the glutathione system. For tGSH, GSH, and GSSG, the comparison between the control and Ectoine 40 mg/kg–treated groups demonstrated negligible effect sizes, with their 95% confidence intervals crossing zero, indicating no statistically significant difference from the control (Fig. 4), thereby confirming complete normalization.

### Effect of Ectoine on Markers of Neuroinflammation

**Fig. 5:**
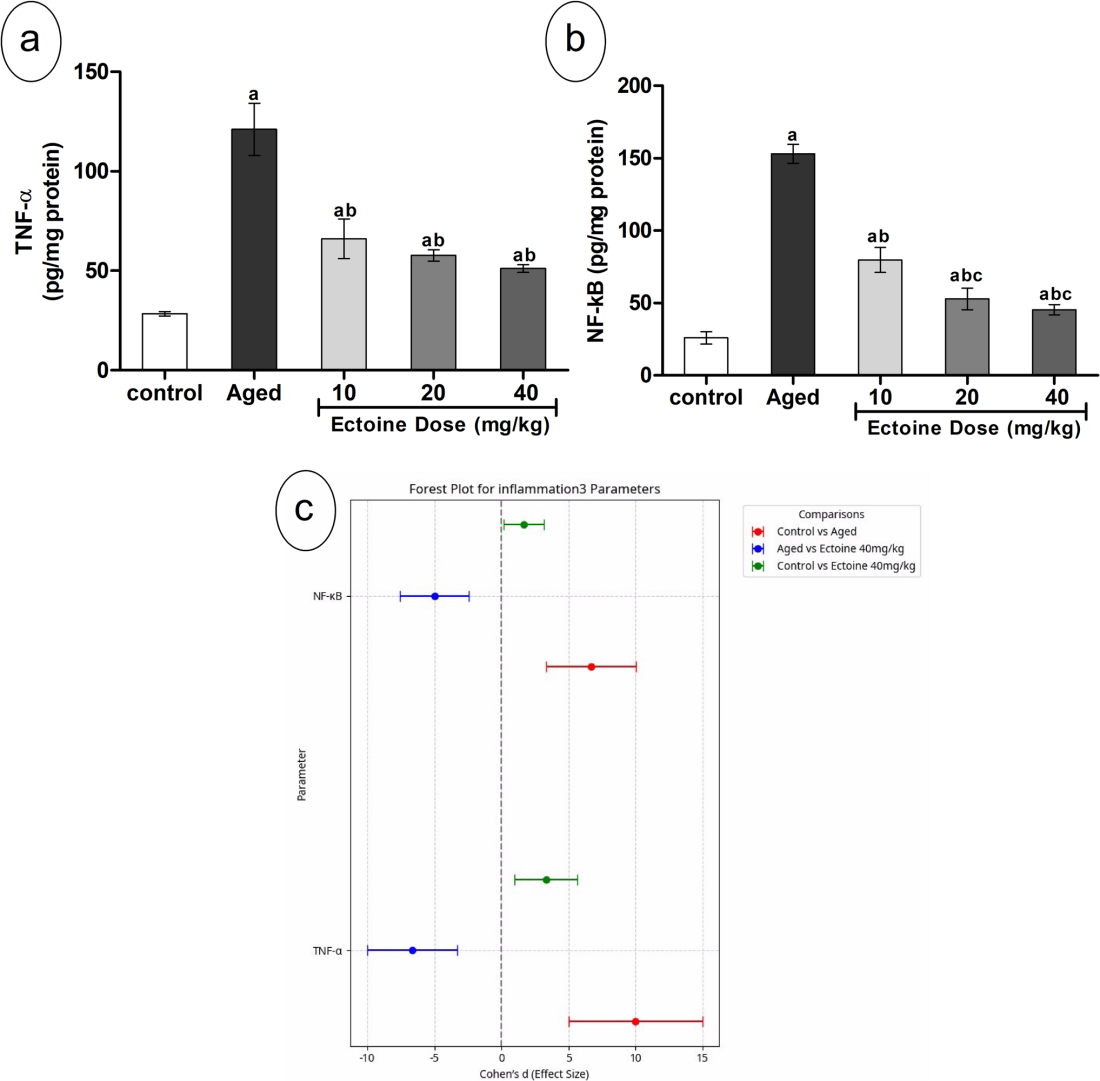
Neuro-inflammation parameters; (**a**) TNF-α, and (**b**) NF-κB in the brain cortex of control rats, aged rats, and aged rats treated with different doses of Ectoine (Data are presented as mean ± SD. *n* = 5. a: significantly different vs. control rats, b: significantly different vs. aged rats, c: significantly different vs. 10 mg/kg Ectoine-treated aged rats, d: significantly different vs. 20 mg/kg Ectoine-treated aged rats by ANOVA followed by Bonferroni post hoc test at *p* < 0.05) (**c**) Effect sizes (Cohen’s *d* ± 95% CI) of aging and Ectoine supplementation on the TNF-α, and NF-κB. (Abbreviations: TNF-α, tumor necrosis factor-alpha; NF-κB, nuclear factor kappa B)

Cortical content of the inflammatory markers, TNF-α and NF-κB, was significantly elevated in aged rats compared to control animals (Fig. 5(a, b)). Ectoine co-supplementation significantly and dose-dependently reduced cortical TNF-α and NF-κB content relative to aged rats. However, despite these reductions, levels remained considerably higher than those in control animals.

Consistent with these findings, the Forest plot (Fig. 5(c)) revealed large effect sizes for both TNF-α and NF-κB when comparing aged rats to controls, indicating a substantial inflammatory response attributable to aging. Furthermore, Ectoine treatment at 40 mg/kg resulted in large effect sizes when comparing the aged group to the treated group, indicating a significant amelioration of these inflammatory markers. However, the comparison between the control group and the Ectoine 40 mg/kg group also showed large effect sizes (Fig. 5(c)), demonstrating that while Ectoine significantly reduced these markers, complete normalization to control levels was not achieved.

### Ectoine Effect on Mitochondrial Homeostasis in The Brain Cortex of Aged Rats

**Fig. 6:**
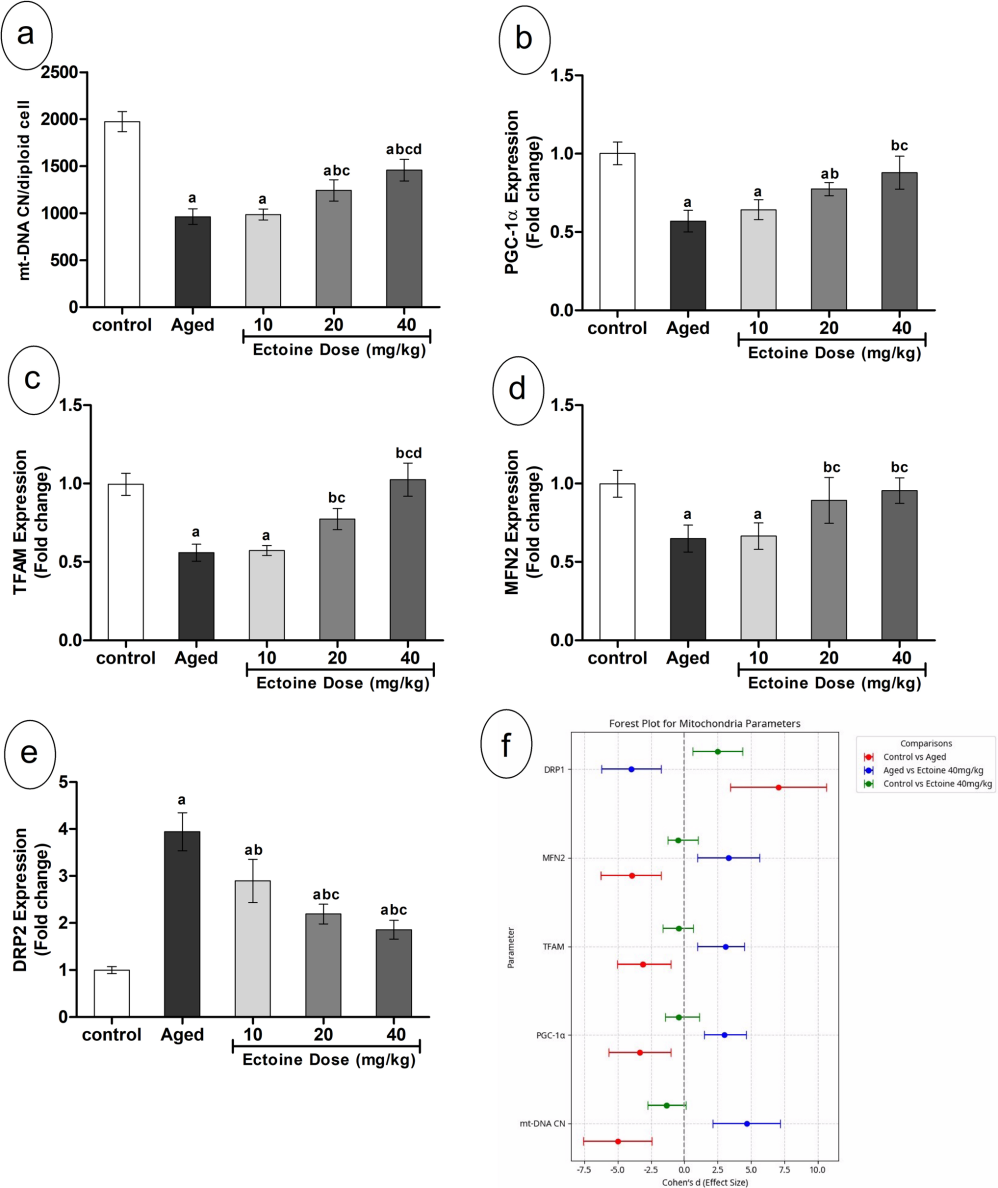
Mitochondrial homeostasis parameters; (**a**) mt-DNA CN, (**b**) PGC-1α, (**c**)TFAM, (**d**) MFN2, and (**e**) DPR1 in the brain cortex of control rats, aged rats, and aged rats treated with different doses of Ectoine (Data are presented as mean ± SD. *n* = 5. a: significantly different vs. control rats, b: significantly different vs. aged rats, c: significantly different vs. 10 mg/kg Ectoine-treated aged rats, d: significantly different vs. 20 mg/kg Ectoine-treated aged rats by ANOVA followed by Bonferroni post hoc test at *p* < 0.05) (f) Effect sizes (Cohen’s *d* ± 95% CI) of aging and Ectoine supplementation on mt-DNA CN, PGC-1α, TFAM, MFN2, and DPR1. (Abbreviations: mt-DNA CN, mitochondrial DNA copy number; PGC-1α, peroxisome proliferator–activated receptor gamma coactivator 1-alpha; TFAM, mitochondrial transcription factor A; MFN2, mitochondrial fusion protein 2; DRP1, Dynamin-related protein 1)

In the brain cortex of aged rats, mitochondrial DNA copy number (mt-DNA CN) exhibited a significant decline of approximately 50% compared to controls. This reduction was accompanied by a marked suppression of gene expression for PGC-1α, TFAM, and MFN2. Conversely, the expression of DRP1, a mitochondrial fission marker, showed significant upregulation (Fig. 6(a–e)). Co-supplementation with Ectoine significantly and dose-dependently increased cortical mt-DNA CN and upregulated the expression of PGC-1α, TFAM, and MFN2 in d-galactose-treated rats. In contrast, DRP1 expression was significantly downregulated in rats co-supplemented with Ectoine in a dose-dependent manner. The most beneficial effects across these parameters were observed at the 40 mg/kg Ectoine dose (Fig. 6(a–e)).

Analysis of the Forest plot (Fig. 6(f)) for all mitochondrial parameters (mt-DNA CN, PGC-1α, TFAM, MFN2, DRP1) revealed substantial dysregulation in aged rats. Large effect sizes indicated significant reductions in markers of mitochondrial biogenesis (PGC-1α, TFAM), fusion (MFN2), and a marked increase in the fission marker (DRP1). Ectoine treatment at 40 mg/kg substantially ameliorated these changes. For all mitochondrial parameters, the comparison between control and Ectoine 40 mg/kg–treated groups demonstrated negligible effect sizes, with their 95% confidence intervals crossing zero, indicating no statistically significant difference from the control (Fig. 6(f)). This finding confirms the complete normalization of these mitochondrial parameters to control levels.

### Ectoine Effects on Apoptotic Markers

**Fig. 7:**
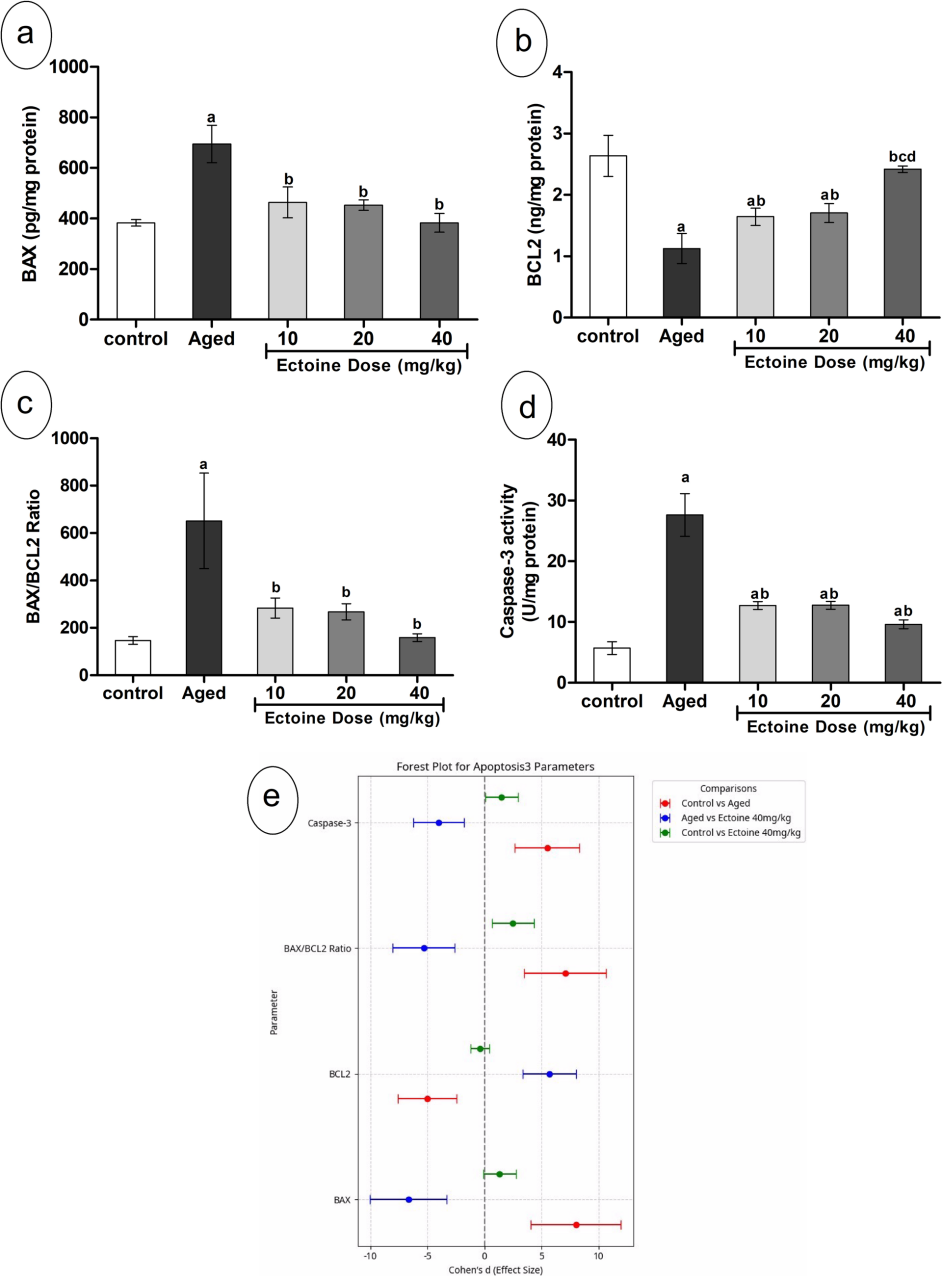
Apoptotic markers; (**a**) BAX, (**b**) Bcl2, (**c**)BAX/Bcl2 ratio, and (**d**) Caspase 3 activity in the brain cortex of control rats, aged rats, and aged rats treated with different doses of Ectoine (Data are presented as mean ± SD. *n* = 5. a: significantly different vs. control rats, b: significantly different vs. aged rats, c: significantly different vs. 10 mg/kg Ectoine-treated aged rats, d: significantly different vs. 20 mg/kg Ectoine-treated aged rats by ANOVA followed by Bonferroni post hoc test at *p* < 0.05) (**e**) Effect sizes (Cohen’s *d* ± 95% CI) of aging and Ectoine supplementation on BAX, Bcl2, BAX/Bcl2 ratio, and Caspase 3 (Abbreviations: BAX, Bcl-2-associated X protein; Bcl2, B cell lymphoma protein 2)

In the brain cortex of aged rats, BAX content was significantly elevated, while BCL2 content showed a significant decline, resulting in a significant increase in the BAX/BCL2 ratio compared to controls. These abnormalities in apoptotic markers were accompanied by significant activation of caspase-3 (Fig. 7(a–d)). Ectoine co-supplementation significantly and dose-dependently increased cortical BCL2 content while decreasing BAX content and the BAX/BCL2 ratio. Notably, all three of these parameters (BAX, BCL-2, and BAX/BCL2 ratio) were completely normalized with the 40 mg/kg Ectoine dose. Furthermore, caspase-3 activity exhibited a significant dose-dependent reduction in Ectoine-supplemented rats. However, even with the highest dose (40 mg/kg), caspase-3 activity was still noticeably higher than in the control group (Fig. 7(d)).

Analysis of apoptotic markers (Bax, Bcl-2, Bax/Bcl-2 ratio, and Caspase-3 activity) revealed large effect sizes when comparing aged rats to controls, indicating a substantial impact of aging on these apoptotic pathways. Ectoine treatment effectively ameliorated these changes. For Bax, Bcl-2, and the Bax/Bcl-2 ratio, the comparison between the control and Ectoine 40 mg/kg–treated groups demonstrated negligible effect sizes, with their 95% confidence intervals crossing zero, thereby confirming complete normalization. Conversely, for caspase-3 activity, the comparison between the control and Ectoine 40 mg/kg–treated groups still showed a large effect size, consistent with its incomplete normalization to control levels (Fig. 7€).

### Effect of Ectoine on Autophagic Markers

**Fig. 8:**
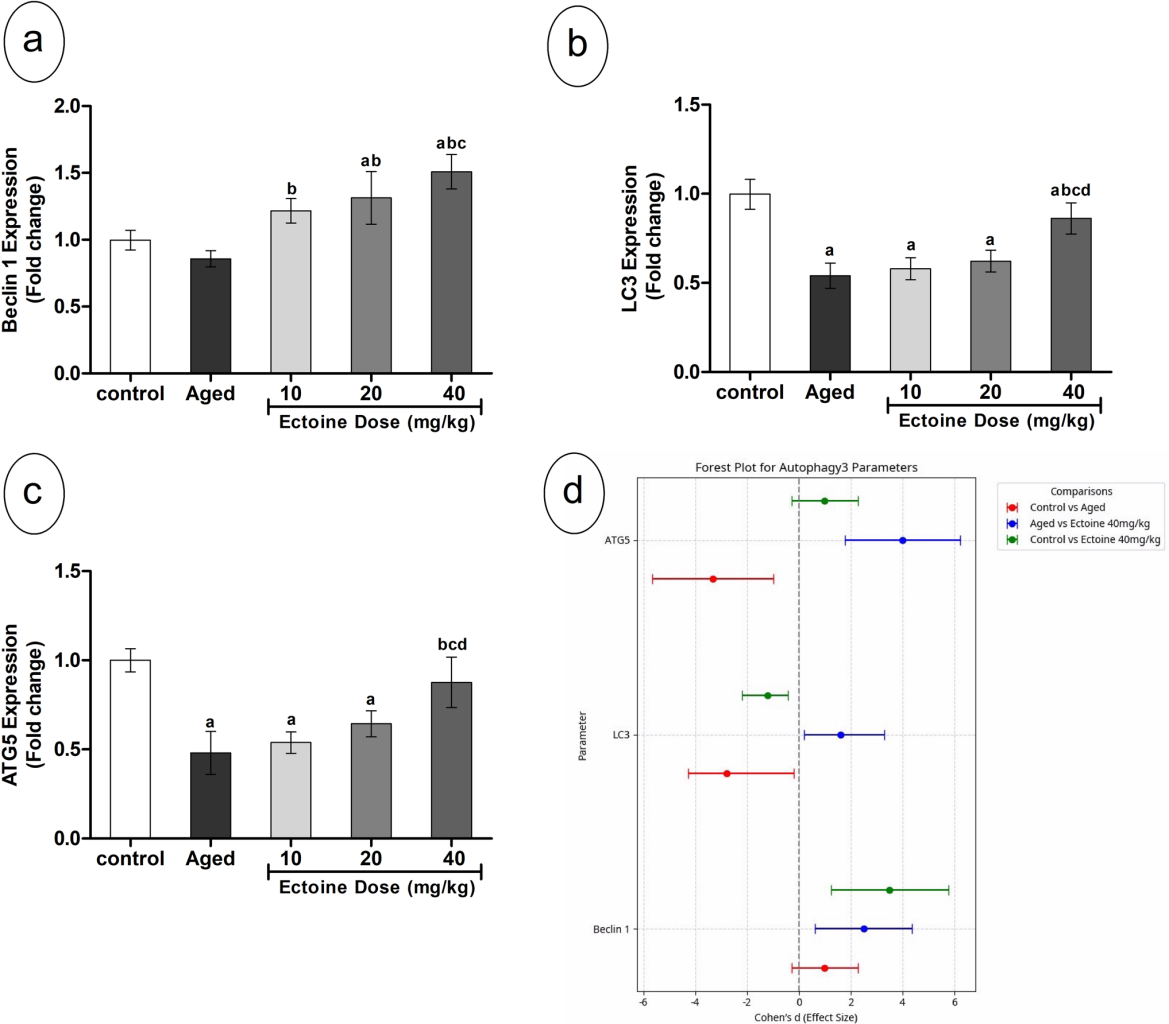
Autophagy parameters; (**a**) Beclin 1, (**b**) LC3, and (**c**) ATG5 in the brain cortex of control rats, aged rats, and aged rats treated with different doses of Ectoine (Data are presented as mean ± SD. *n* = 5. a: significantly different vs. control rats, b: significantly different vs. aged rats, c: significantly different vs. 10 mg/kg Ectoine-treated aged rats, d: significantly different vs. 20 mg/kg Ectoine-treated aged rats by ANOVA followed by Bonferroni post hoc test at *p* < 0.05) (**d**) Effect sizes (Cohen’s *d* ± 95% CI) of aging and Ectoine supplementation on Beclin 1, LC3, and ATG5 (Abbreviations: LC3, microtubule-associated protein 1A/1B-light chain 3; ATG5, autophagy protein 5)

As presented in Fig. 8, cortical gene expression of Beclin 1 remained unchanged in aged rats compared to controls, while the expression of LC3 and ATG5 was significantly suppressed (by approximately 50%). Ectoine co-supplementation resulted in a dose-dependent upregulation of Beclin 1 expression, which was elevated compared to both control and aged rats (Fig. 8(a)). The expression of LC3 and ATG5 showed no significant changes with lower Ectoine doses (10 and 20 mg/kg), but a significant upregulation was observed in rats supplemented with the highest dose (40 mg/kg) (Fig. 8(b, c)).

Analysis of the Forest plot (Fig. 8(d)) indicated that LC3 and ATG5 exhibited large negative effect sizes in aged rats compared to controls, reflecting their significant suppression. In contrast, Beclin 1 showed a negligible effect size, with its 95% confidence interval crossing zero, consistent with aged rats showing no noticeable alteration in comparison to controls. The Ectoine 40 mg/kg group demonstrated substantial positive effect sizes for LC3 and ATG5 when compared to the aged group, indicating significant amelioration and an approach towards control levels (Fig. 8(d)).

### Effects of Ectoine on Amyloidogenic Markers

**Fig. 9:**
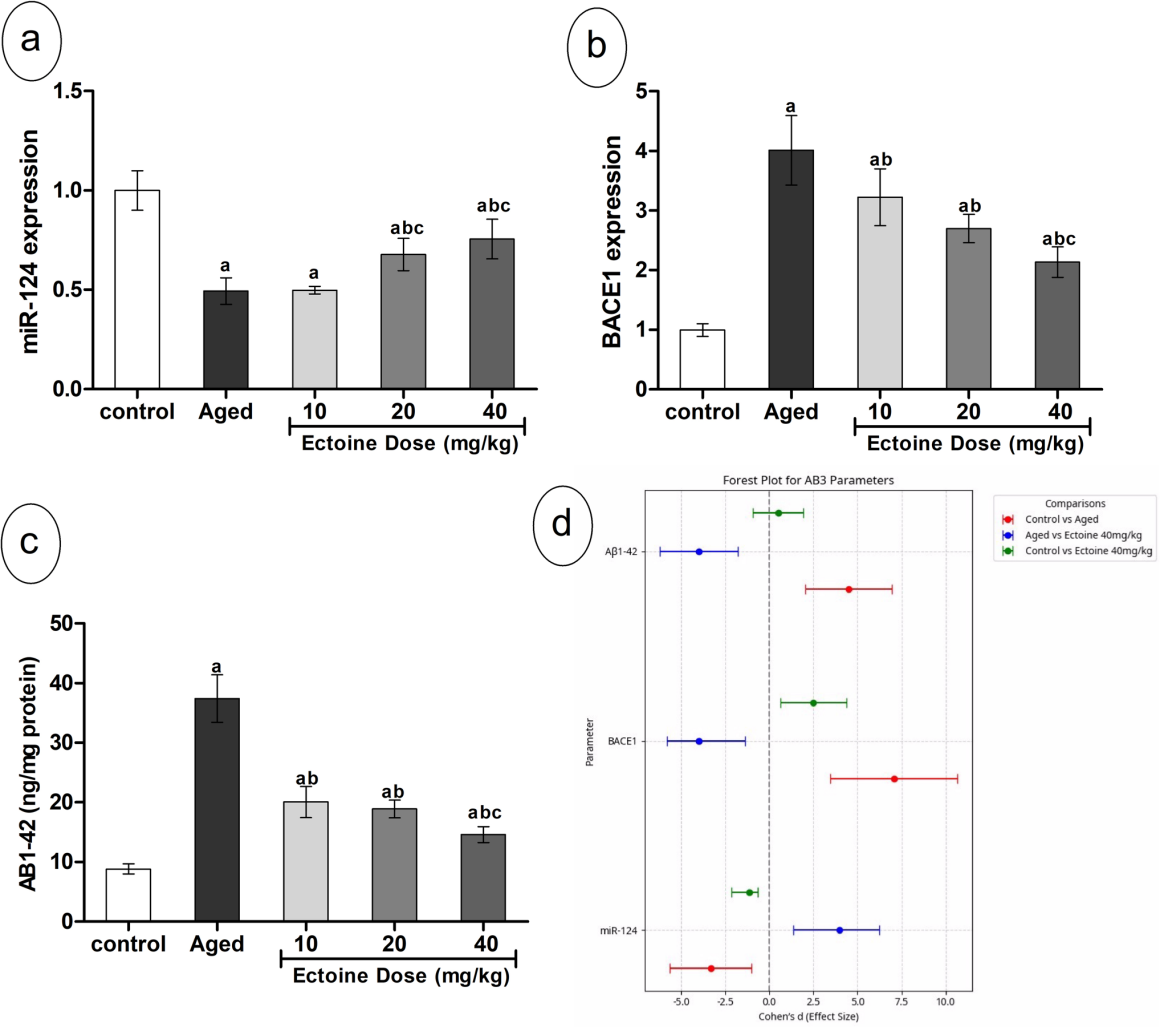
Amyloidogenic parameters; (**a**) miR-124, (**b**) BACE1, and (**c**)Aβ1–42 in the brain cortex of control rats, aged rats, and aged rats treated with different doses of Ectoine (Data are presented as mean ± SD. *n* = 5. a: significantly different vs. control rats, b: significantly different vs. aged rats, c: significantly different vs. 10 mg/kg Ectoine-treated aged rats, d: significantly different vs. 20 mg/kg Ectoine-treated aged rats by ANOVA followed by Bonferroni post hoc test at *p* < 0.05) (**d**) Effect sizes (Cohen’s *d* ± 95% CI) of aging and Ectoine supplementation on miR-124, BACE1, and Aβ1–42 (Abbreviations: BACE1, β-site APP cleaving enzyme 1; Aβ 1–42, β-amyloid peptide 1–42)

In aged rats, cortical Aβ−42 peptide content was significantly elevated compared to controls. This was accompanied by a significant upregulation of BACE1 expression (Fig. 9(a, b)). These amyloidogenic abnormalities were associated with significant downregulation of miR-124. However, Ectoine co-supplementation resulted in a significant dose-dependent upregulation of miR-124 compared to aged rats. Conversely, Ectoine co-supplementation significantly and dose-dependently downregulated BACE1 expression and decreased Aβ−42 content. Despite these reductions, Aβ−42 levels remained considerably higher than those in control animals, as shown in Fig. 9(c).

Analysis of the Forest plot (Fig. 9(d)) for amyloidogenic markers (miR-124, BACE1, Aβ1–42) revealed large effect sizes when comparing aged rats to controls, indicating substantial dysregulation of the amyloidogenic pathway due to aging. Ectoine treatment significantly ameliorated these changes. Specifically, while the Ectoine 40 mg/kg group demonstrated significant improvement in all markers, the comparison between the control and Ectoine 40 mg/kg–treated groups for Aβ1–42 still yielded a large effect size. This finding is consistent with Aβ1–42’s incomplete normalization to control levels, as its 95% confidence interval did not cross zero (Fig. 9(d)).

### The Correlation Results

Correlation analysis (Table 3) revealed significant positive correlations between the apoptotic BAX/BCL2 ratio and both oxidative stress markers (MDA and 8-OHdG) and neuroinflammation markers (TNF-α, NF-κB). Conversely, the Bax/Bcl-2 ratio exhibited significant negative correlations with autophagic markers (Beclin 1, ATG5, LC3), mitochondrial DNA copy number (mtDNA CN), and components of the antioxidant system (NRF2, HO-1, GSH). Additionally, the amyloidogenic markers (Aβ1–42, BACE1) displayed significant negative correlations with miR-124.

**Table 3:** Correlation coefficient between different parameters in aged rats, untreated, and Ectoine-treated

	miR-124	Aβ1–42	SIRT-1	BAX/Bcl2	Beclin1	ATG5	LC3	NRF2	HO-1	mtDNA CN
MDA	− 0.639**	0.835**	− 0.793**	0.848**	− 0.895**	− 0.781**	− 0.682**	− 0.700**	− 0.596**	− 0.801**
8-OHdG	− 0.720**	0.836**	− 0.935**	0.780**	− 0.847**	− 0.673**	− 0.668**	− 0.899**	− 0.667**	− 0.810**
TNF-α	− 0.592**	0.877**	− 0.832**	0.789**	− 0.881**	− 0.524*	− 0.604**	− 0.909**	− 0.791**	− 0.623**
NF-κB	− 0.673**	0.963**	− 0.906**	0.874**	− 0.862**	− 0.640**	− 0.614**	− 0.899**	− 0.809**	− 0.711**
GSH/GSSG	0.842**	0.817**	0.938**	− 0.762**	0.836**	0.766**	− 0.857**	0.821**	0.620**	0.807**
TFAM	0.774**	− 0.629**	0.812**	− 0.551**	0.738**	0.723**	0.874**	0.697**	0.464*	0.855**
DRP1	− 0.709**	0.876**	− 0.950**	0.791**	− 0.787**	− 0.667**	− 0.679**	− 0.874**	− 0.711**	− 0.713**
MFN2	0.833**	− 0.616**	0.667**	− 0.580**	0.681**	0.785**	0.557**	0.666**	0.238	0.696**
miR-124	1	− 0.613**	0.716**	− 0.544**	− 0.631**	0.635**	0.697**	0.749**	0.303	0.717**
Aβ1–42	− 0.613**	1	− 0.836**	0.933**	− 0.799**	− 0.696**	− 0.577**	− 0.741**	− 0.827**	− 0.658**
BACE1	− 0.745**	0.772**	− 0.889**	0.649**	− 0.789**	− 0.668**	− 0.818**	− 0.840**	− 0.676**	− 0.715**
SIRT-1	0.716**	− 0.836**	1	− 0.778**	0.810**	0.738**	0.786**	0.857**	0.709**	0.799**
BAX/Bcl2	− 0.544*	0.933**	− 0.778**	1	− 0.761**	− 0.717**	− 0.479**	− 0.651**	− 0.748**	− 0.661**
Beclin1	0.631**	− 0.799**	0.810**	− 0.761**	1	0.672**	0.679**	0.840**	− 0.586**	0.621**
ATG5	0.635**	− 0.696**	0.738**	− 0.717**	0.672**	1	0.665**	0.480*	0.383	0.826**
LC3	0.697**	− 0.577**	0.786**	− 0.479**	− 0.679**	0.665**	1	0.618**	0.506*	0.755**
mtDNA CN	0.717**	− 0.658**	0.799**	− 0.661**	0.621**	0.826**	0.755**	0.602**	0.507*	1

Correlation study was checked by Pearson correlation. *Significant correlation at *p* < 0.05; **significant correlation at *p* < 0.01

### Histological Studies

**Fig. 10:**
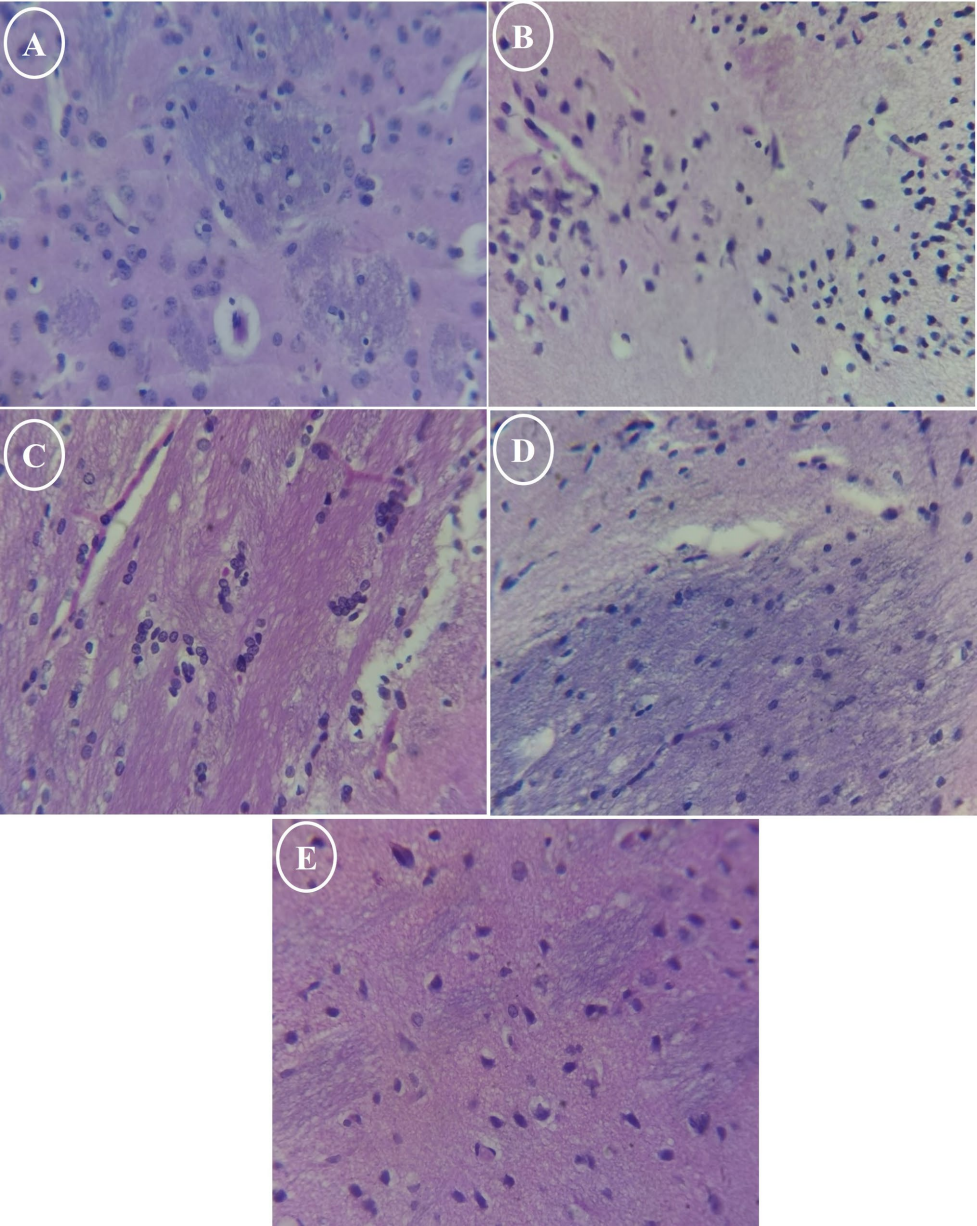
Representative photomicrographs of H and E-stained sections of the rat’s brain cortex from control rats, aged rats, and aged rats treated with different doses of Ectoine. **A** Rats from the control group, **B** rats from the aging group, **C** rats from the Ectoine 10 mg/kg–treated group, **D** rats from the Ectoine 20 mg/kg–treated group, and **E** rats from the Ectoine 40 mg/kg–treated group (× 100)

Histological examination of brain sections from control rats revealed intact nerve fibers, a normal appearance of pigmented substantia nigra neurons, and healthy oligodendrocytes (Fig. 10A). In contrast, brain sections from accelerated-aged rats exhibited widespread neuropathological changes, including vacuolation, spongiosis, and cellular degeneration. These changes were further characterized by mild neuronal damage accompanied by gliosis (evidenced by pyknotic glial cells), infiltration of inflammatory cells, and early neuronal necrosis, resulting in noticeable disarray in cellular distribution (Fig. 10B). Ectoine co-supplementation induced a dose-dependent amelioration of the aforementioned histological abnormalities observed in aged rats. While the overall severity of vacuolation, spongiosis, and cellular degeneration was markedly reduced, residual signs such as slight chromatolysis of nuclear material and persistent mild neuronal damage with associated gliosis remained evident (Fig. 10C–E).

## Discussion

Addressing anti-aging agents to protect the brain from aging is of great importance because targeting aging pathways preserves brain function, combats neurodegenerative diseases, and extends health span, making it vital for individual and public health. Ectoine has been demonstrated to have antioxidant, anti-inflammatory, anti-apoptotic, and neuroprotective properties and is considered a potential anti-aging agent [35]. The present study substantially extends the current understanding of Ectoine’s neuroprotective potential by exploring its oral administration, a previously underexplored route within the context of d-galactose-induced brain aging. This widely accepted preclinical model recapitulates key features of neurodegeneration. This is the first report to demonstrate that oral Ectoine has the potential to: restore mitochondrial homeostasis by modulating PGC-1α, TFAM, DRP1, and MFN2 gene expression, enhance autophagic flux via Beclin-1, LC3, and ATG5 upregulation, and attenuate the amyloidogenic pathway through miR-124/BACE1/Aβ1–42 axis modulation—a novel mechanistic insight not previously attributed to Ectoine in the brain.

The D-gal-induced brain aging model was selected for this research due to its established utility in simulating brain senescence, enabling the investigation of fundamental aging processes and the efficacy of anti-aging treatments [2]. At the histological level, our data from this model revealed vacuolation, spongiosis, cellular degeneration and necrosis, and prominent gliosis. These findings are consistent with previous studies demonstrating that long-term d-galactose exposure induces increased oxidative stress and reduced antioxidant activity, thereby mimicking the degenerative and pathological changes observed in the aging brain [36, 37]. Importantly, Ectoine co-supplementation resulted in a dose-dependent amelioration of these histological abnormalities in the aged rats.

In the present study, the brain cortex of aged rats exhibited significant elevations in markers of oxidative damage: MDA (lipid peroxidation index), 8-OHdG (DNA oxidative marker), and GSSG (oxidized glutathione). These results align with previous clinical studies [38, 39]. This oxidative stress was coupled with compromised antioxidant defense systems in the aged rat brain cortex, indicated by marked declines in GSH levels, redox ratio, and the expression of NRF2, HO-1, and SIRT1. SIRT1 plays a crucial role in aging by regulating genes involved in autophagic signaling and apoptotic pathways. Its reduced protein levels in aged cells and organs are often associated with senescence, dendritic loss, and impaired neurotransmitter production [8]. The NRF2/HO-1 pathway is a prominent endogenous antioxidant defense mechanism, with NRF2 serving as the primary transcriptional regulator of endogenous antioxidants [11]. Our findings corroborate previous studies demonstrating downregulated NRF2 and HO-1 expression in response to D-gal-induced oxidative stress in aged mice [40].

A compelling body of evidence implicates impaired NRF2 pathway expression in aging and age-associated neurodegenerative diseases (as reviewed in [12]). Consequently, therapeutic agents that target the NRF2/ARE/HO-1 pathway show promise in protecting against or alleviating age-related pathologies. For instance, Chen et al. reported that Leonurine exhibits anti-aging effects, potentially mediated by the upregulation of the NRF2 signaling pathway [41, 42]. In the present study, Ectoine co-administration with D-gal dose-dependently restored the levels of NRF2, HO-1, and SIRT1 proteins in the brain cortex. These results indicate that Ectoine can protect the brain from D-gal-induced oxidative stress and aging by modulating the NRF2 and SIRT1 signaling pathways. SIRT1 is well established for its role in prolonging lifespan through the reduction of oxidative stress and suppression of pro-inflammatory cytokine expression [8, 43, 44]. Furthermore, SIRT1 can interact with and modulate several autophagy-related genes [45]. The pivotal role of SIRT1 in Ectoine’s anti-aging effects was further underscored by the positive correlation observed between SIRT1 protein levels and the expression of NRF2, mt-DNA CN, miR-124, and LC3 in the Ectoine-co-supplemented aged rats. This suggests a mechanistic link between elevated SIRT1 and the observed improvements in apoptotic, autophagic, and amyloidogenic pathways.

Consistent with previous reports, significant declines in acetylcholine (ACh) and dopamine levels were observed in the brain cortex of aged rats, a deficit that contributes to cognitive dysfunction [46]. Ectoine co-supplementation significantly attenuated the D-gal-induced reduction of ACh in a dose-dependent manner. This suggests that Ectoine can improve cholinergic system function and offer neuroprotection against age-related changes in the brain.

In the d-galactose-induced aging model, rats exhibited a marked induction of the amyloidogenic pathway, characterized by elevated Aβ1–42 levels, enhanced BACE1 expression, and suppressed expression of the protective microRNA-124 (miR-124). Consistent with existing literature, miR-124 is known to be downregulated by pro-inflammatory stimuli. In our model, this was evidenced by the significant elevation of brain neuroinflammatory markers, specifically NF-κB and TNF-α. This suppression of miR-124 consequently led to the induction of BACE1, thereby triggering the amyloidogenic pathway. Therefore, targeting miR-124 for upregulation represents a promising strategy to suppress this pathway during aging [6].

In the current study, Ectoine supplementation significantly upregulated miR-124 expression, which was concomitantly associated with a dose-dependent reduction in BACE1 expression and Aβ1–42 levels. The pivotal role of miR-124 in mediating Ectoine’s anti-amyloidogenic effects is further supported by its negative correlation with BACE1, Aβ1–42, NF-κB, and TNF-α. These correlative patterns suggest that these are potential direct or indirect targets of miR-124. Therefore, future studies employing techniques such as qPCR and luciferase reporter assays are warranted to confirm direct regulatory interactions between miR-124 and these specific genes. Furthermore, miR-124’s involvement in other pathogenic pathways within the brain is suggested by its negative correlation with the pro-apoptotic protein BAX, and positive correlations with the autophagic marker LC3, MFN2, and mtDNA CN. These findings collectively suggest a broader role for miR-124 in promoting brain autophagy and mitochondrial homeostasis. The observed modulation of miR-124 expression by Ectoine could be linked to Ectoine’s broader antioxidant and anti-inflammatory properties, potentially influencing the epigenetic regulation of miRNA expression. This is plausible given that oxidative stress and neuroinflammation are known to downregulate miR-124 in aging models [47].

To our knowledge, no prior studies have specifically investigated Ectoine’s modulation of miR-124 in the context of brain aging or any other biological model. While our results are consistent with known regulatory patterns in brain aging, they do not definitively prove that Ectoine acts directly upstream of miR-124. Although Ectoine supplementation resulted in a dose-dependent upregulation of miR-124 and a concomitant downregulation of BACE1 and Aβ1–42, we acknowledge that this relationship remains correlative. The current study does not establish whether Ectoine directly regulates miR-124 at the transcriptional or post-transcriptional level. The exact molecular mechanisms by which Ectoine affects miR-124 (e.g., via specific transcription factors or epigenetic modifications) warrant further investigation and will be a focus of future studies. Future studies are also needed to explore the effects of miR-124 overexpression and knockdown in neuronal cell lines or in vivo rat models to conclusively determine whether miR-124 is essential for Ectoine’s observed protective effects.

Proper mitochondrial homeostasis is a vital requirement for a healthy brain as it is a highly metabolic organ, and any mitochondrial abnormality is considered a primary risk factor in the development of aging-related neurodegenerative diseases. Mitochondrial dysfunction is a hallmark of aging, and accumulated mitochondrial DNA damage, ROS overproduction, and declined quality control mechanisms drive brain aging [18]. Our results confirmed the dysregulated mitochondrial homeostasis in the brain cortex of aged rats as indicated by impaired mitochondrial biogenesis (decline in mtDNA CN, suppressed expression of PGC-1α and TFAM), and fusion (suppressed MFN2), and induced fission (enhanced expression of DRP1). Extensive studies documented that the mitochondrial biogenesis genes exhibited significant downregulation in aging [48]. Also, the D-gal-induced aging model showed enhanced DRP1 expression and suppressed MFN2 expression [49]. These dysregulations in mitochondrial homeostasis may be a consequence of D-gal-induced oxidative stress, which can promote mitochondrial fission. Impaired equilibrium of mitochondrial fission and fusion has been linked to aging and aging-related neurodegenerative disorders [50–52].

Ectoine co-supplementation with D-gal significantly and dose-dependently restored the mitochondrial homeostasis through upregulated expression of PGC-1α, TFAM, and MFN2 and downregulation of DRP1 expression, which resulted in maintaining the cortical mtDNA CN close to the normal value with the highest dose of Ectoine used (40 mg/kg). The protective role of Ectoine on mtDNA against radiation damage was previously reported [53, 54]. Also, Ectoine can protect the native structures of proteins, lipid membranes, and mtDNA [55, 56].

It is commonly recognized that autophagy and mitophagy play critical roles in mitochondrial quality control, particularly in the presence of mitochondrial damage or malfunction. Consequently, autophagy deficiency can lead to the accumulation of damaged mitochondria, thereby triggering oxidative damage and apoptosis [49]. In the present study, to verify the functional status of autophagy, we assayed the expression of Beclin 1, LC3, and ATG5 in the brain cortex and we found that the mitochondrial abnormalities in aged rats were associated with impaired autophagy as indicated by a decline in the autophagic genes LC3 and ATG5 with no significant changes in Beclin 1 expression, which may imply that D-gal-induced brain aging is associated with impaired autophagy progression without changes in the initiation through Beclin1 which is in agreement with a previous study. Increased production of NF-κB-driven anti-apoptotic Bcl-2 proteins can inhibit Beclin 1-dependent autophagy [2]. Further supporting this interpretation, correlation analyses revealed that NF-κB was negatively correlated with all assessed autophagic markers: Beclin 1, LC3, and ATG5. However, Ectoine supplementation significantly and dose-dependently upregulated the expression of these autophagic markers, suggesting activation of both autophagy initiation and progression. Similarly, prior research has reported that Ectoine administration effectively enhanced autophagic efficiency in a mouse model of Duchenne muscular dystrophy (DMD) by upregulating the autophagic proteins LC3 and ATG5 [57].

Increased apoptosis is a hallmark of brain aging, implicated in various age-related pathological conditions and serving as a common marker of neuronal damage [14, 19]. Mitochondria play a central role in both intrinsic and extrinsic apoptotic pathways, with key regulators including the pro-apoptotic protein BAX, the anti-apoptotic protein BCL-2, and caspase-3. BAX and BCL-2 are upstream regulators of caspase-3 activation [2]. Consequently, the balance between BAX and BCL-2, often expressed as the BCL-2/BAX ratio, is a critical determinant of cellular apoptosis progression. In the present study, aged rats exhibited an elevated BAX/BCL-2 ratio concurrent with enhanced caspase-3 activity in the brain cortex, consistent with previous findings [58]. Ectoine supplementation restored the BAX/BCL-2 ratio and normalized caspase-3 activity in the brain cortex, compared to the D-gal-treated control group. These results suggest that Ectoine enhances anti-apoptotic capacity by modulating the balance between pro-apoptotic and pro-survival signals in the brains of aged rats.

The observed multi-target effects of Ectoine strongly suggest a multi-mode mechanism of action. Ectoine is conventionally recognized as a kosmotrope and protein stabilizer, known for its ability to enhance hydrophobic interactions, facilitate protein folding, and stabilize cell membranes [26]. These properties may underline its antioxidant and anti-apoptotic activities. Furthermore, Ectoine’s influence may extend to epigenetic regulation. Prior reports indicate that Ectoine can modulate global DNA methylation and regulate the expression of genes involved in epigenetic modification [54]. This epigenetic modulation could account for its broader effects on the expression of various genes.

Ectoine’s effects on the various biomarkers investigated exhibited an inconsistent dose–response pattern. While several biomarkers (e.g., miR-124, BACE1, Aβ1–42, LC3, ATG5, MFN2, DRP1, mtDNA copy number, PGC-1α, TFAM, BCL2) showed clear dose-dependent trends, with higher doses (20–40 mg/kg) yielding optimal effects, other biomarkers (e.g., NRF2, HO-1, NF-κB, BAX, and caspase-3) displayed non-linear or plateau effects, particularly at the highest dose (40 mg/kg). This observed inconsistency may reflect the complex interplay of pathways involved in brain aging, where Ectoine’s influence on redox, mitochondrial, and inflammatory pathways might reach saturation at higher doses or vary due to biomarker-specific sensitivities. The Ectoine doses administered were initially selected based on safety and efficacy data derived from non-neurological studies and were adjusted considering oral solubility and systemic absorption. However, without concurrent pharmacokinetic validation, the effective brain bioavailability of Ectoine in this model remains unconfirmed. Consequently, further pharmacokinetic studies encompassing a broader dose range (e.g., 5–100 mg/kg) are essential to optimize future dosing strategies by thoroughly assessing Ectoine’s bioavailability and brain penetration in rats.

While we acknowledge the modest sample size of *n* = 5 per group (selection guided by IACUC ethical constraints), the consistent observation of large effect sizes, coupled with relatively narrow CIs for these effects, provides compelling evidence for the robustness and biological significance of our findings. Effect sizes offer a measure of the practical importance of an observed difference, independent of sample size, and our results indicate that the observed effects are not only statistically significant but also biologically meaningful.

The current study serves as an exploratory investigation, generating mechanistic hypotheses concerning Ectoine’s systemic effects on brain aging pathways. Although our findings suggest tissue-level neuroprotection via Ectoine’s modulation of oxidative stress, mitochondrial function, apoptosis, autophagy, and amyloidogenesis, the absence of cell-type-specific resolution precludes definitive attribution of these effects to neuronal, astrocytic, or microglial populations. Given the differential vulnerability and signaling roles of these cell types in brain aging, future work employing immunophenotyping, fluorescence-based co-localization, cell sorting, or in situ hybridization will be critical to identify Ectoine’s primary cellular targets. Additionally, subsequent studies should investigate whether Ectoine synergizes with other neuroprotective agents, such as astaxanthin, resveratrol, or luteolin, through co-administration experiments to assess potential additive or antagonistic effects on cognitive outcomes and molecular pathways. Such experiments will enhance the translational relevance of our findings and are slated for further investigation.

While the D-gal-accelerated aging rat model recapitulates several molecular and histological hallmarks of human brain aging, species-specific differences in drug metabolism, blood–brain barrier transport, and neuroimmune regulation may influence its translational relevance. Consequently, future preclinical bridging studies in higher-order species, coupled with clinical pharmacokinetic profiling, will be essential to confirm adequate brain exposure and validate efficacy in humans.

Collectively, Ectoine co-supplementation during D-gal induced accelerated aging in rats significantly attenuated the main features of brain aging across histological, biochemical, and molecular levels. Ectoine exerted its anti-aging effects through antioxidant, anti-inflammatory, anti-apoptotic, autophagy-promoting, and enhanced mitochondrial function. Therefore, given its inherent biocompatibility and non-interference with cellular biochemistry and metabolism, even at high molar concentrations [5, 27]. Ectoine holds significant promise for positively impacting the brain health of elderly individuals, a potential strongly supported by the findings of this study.

## Data Availability

No datasets were generated or analysed during the current study.
